# Biliary tumorigenic effect on hypopharyngeal cells is significantly enhanced by pH reduction

**DOI:** 10.1002/cam4.2194

**Published:** 2019-06-07

**Authors:** Sotirios G. Doukas, Bruno Cardoso, Jacob I. Tower, Dimitra P. Vageli, Clarence T. Sasaki

**Affiliations:** ^1^ The Yale Larynx Laboratory Department of Surgery Yale School of Medicine New Haven Connecticut USA

**Keywords:** acid, bile acids, hypopharyngeal cancer, laryngopharyngeal reflux, NF‐κB, pH

## Abstract

Biliary reflux has been considered a potential risk factor in upper aerodigestive tract malignancies. It is not yet clearly known how pH affects the bile‐induced activation of NF‐κB and its related oncogenic pathway previously linked to hypopharyngeal carcinogenesis. In this study, repetitive applications of conjugated primary bile acids with unconjugated secondary bile acid, deoxycholic acid (DCA), on human hypopharyngeal primary cells reveal that strongly acidic pH (4.0) optimally enhances the tumorigenic effect of bile, by inducing activation of NF‐κB, STAT3 nuclear translocation, bcl‐2 overexpression and significant overexpression of the oncogenic mRNA phenotype, compared to weakly acidic pH (5.5) or neutral pH (7.0). As the pH becomes less acidic the partially activated primary bile acids and activated DCA begin to exert their influence; however, with significantly less intensity compared to bile acids at strongly acidic pH. Our findings suggest that biliary tumorigenic effect is strongly pH dependent. Controlling pH during reflux events may be therapeutically effective in reducing the potential risk of bile‐induced hypopharyngeal cancer.

## 1 INTRODUCTION

According to the American Cancer Society approximately 3000 patients will be diagnosed with hypopharyngeal cancer in 2019, while only 53% of patients diagnosed with a malignant neoplasm will survive up to 5 years.^1^ Among other risk factors, such as tobacco and alcohol,^2^, ^3^ laryngopharyngeal reflux disease (LPR), has also been associated with hypopharyngeal neoplasms as gastroesophageal reflux disorder (GERD) has been epidemiologically linked to upper aerodigestive tract malignancies.^4^ Recent epidemiologic evidence suggests that reflux disease is an independent risk factor for laryngopharyngeal carcinoma.^5^ However, the exact mechanism by which reflux promotes risk of malignancy remains unclear.

In general, during reflux events, gastric and duodenal components leak through an incompetent lower esophageal sphincter and come into contact with the esophageal mucosa, which commonly causes symptoms of heartburn and dysphagia. Similarly, reflux of gastric and duodenal components reaching the laryngopharynx is defined as LPR and commonly presents with laryngeal symptoms.^6^ Clinically, intraesophageal pH monitoring has been used extensively for the identification of reflux episodes. Although pH varies during gastroesophageal reflux, pH 4.0 has generally been accepted as the cut‐off value for documented reflux events^7^ since patients usually experience heartburn symptoms at this level of acidity. In 50%‐86% of patients with GERD, bile is detected within esophageal aspirates.^8^, ^9^, ^10^, ^11^ Also, others have detected bile 15% of the time when the pH is <4.0, 19% of the time when the pH is between 4.0 and 7.0, and 6% of the time when the pH exceeds 7.0.^12^

Bile found in esophageal aspirates usually consists of primary bile acids conjugated with glycine or taurine, but secondary, unconjugated bile acids, such as deoxycholic acid (DCA), can also be identified.^10^, ^13^ Depending on the pH, these bile acids exist in either ionized or un‐ionized form. In theory, un‐ionized bile acids tend to have a more harmful effect since they are better able to penetrate or interact with the cell membranes of epithelial cells.^14^ At the pH range between 3.0 and 5.5, bile acids may exist in both ionized and un‐ionized forms (taurocholic acid and taurodeoxycholic acid: pKa 1.8‐1.9; glycocholic acid and glycodeoxycholic acid: pKa 4.3‐5.2).^14^ At a lower pH (pH < 3.0), all bile acids precipitate, and at a higher pH, between 5.5 and 7.0, most conjugated primary bile acids remain ionized and therefore inactive. On the other hand, DCA (pKa 5.5‐6.5), previously shown to have carcinogenic potency in the colon through NF‐κB activation, is un‐ionized and therefore more capable of penetrating the mucosa at a weakly acidic pH range.^15^ At pH 7.0, DCA is predominantly in the ionized form, and therefore considered to be inactive.

The management of GERD and LPR has been focused on the suppression of gastric acid secretion. Proton pump inhibitors are highly effective in the management of acid secretion and have been extensively used in the treatment of GERD and LPR. Although acid suppression has been an effective means of controlling the symptoms of reflux its effectiveness in preventing GERD related malignancies nevertheless remains controversial.^16^

Our previous in vitro and in vivo exploration have shown that the effect of acidic bile (bile at pH equal or <4.0) can lead to a significant transcriptional activation of NF‐κB anti‐apoptotic pathway in exposed human hypopharyngeal primary cells (HHPC)^17^ and in premalignant murine hypopharyngeal mucosa^18^; furthermore, our data suggest that such changes can be effectively prevented by NF‐κB inhibition.^19^, ^20^, ^21^, ^22^ Our previous studies have also shown that bile at neutral pH (7.0) has a significantly less intense effect than acidic bile whereas acid alone, pepsin and other nonspecific stress factors such as highly concentrated glucose do not result in similar preneoplastic molecular events.^17^, ^18^, ^19^, ^20^, ^21^, ^22^, ^23^ These data support the carcinogenic potency of acidic bile, resulting in the subsequent deregulation of genes with anti‐apoptotic or oncogenic functions linked to NF‐κB and related to head and neck squamous cell carcinomas.

Because preliminary data suggest that the biliary oncogenic effect is pH dependent, systematically characterizing this relationship may yield clinically useful information. To accomplish this, we have used our prior in vitro model to explore the effect of bile, in ranges of pH between 4.0 (strongly acidic), 5.5 (weakly acidic) and 7.0 (neutral) on hypopharyngeal cells in activating the transcriptional factor NF‐κB and related oncogenic mRNA phenotypes.

## 2 MATERIALS AND METHODS

### 2.1 Cell culture and treatment conditions

#### 2.1.1 HHPC culture

HHPC were provided by Celprogen Inc., Torrance, CA and cultured as previously described.^17^, ^19^ In detail, The HHPC were plated in noncoated flasks and were grown in Human Hypopharyngeal Normal Cell Culture Media with Serum (Celprogen Inc. CA), at 37°C in humidified air and 5% CO_2_. The HHPC were subcultured and media were gradually replaced by Serum Free Media (Celprogen Inc.), and passed after reaching ~90% confluence, using 0.05% trypsin‐EDTA (Gibco^®^, NY).

#### 2.1.2 Treatment conditions

HHPC (second passage) underwent repetitive exposure to experimental and control media for 7 minutes, two times per day, for 4 days.

Experimental groups included repetitive exposure to (a) bile at pH 4.0 (strongly acidic), (b) bile at pH 5.5 (weakly acidic), and (c) bile at pH 7.0 (neutral), as follows: (a) bile at pH 4.0 (strongly acidic), containing 400 μM of conjugated bile salts mixture (GCA+TCA+GCDCA+TCDCA+GDCA+TDCA, Sigma, St. Louis, MO and Calbiochem, San Diego, CA) at molar concentration (20:3:15:3:6:1) as previously described,^17^ with or without the unconjugated bile salt, DCA, in concentration of 250 μM (VWR International, Alfa Aesar, MA). The concentrations of the bile salts we selected were based on the concentrations found in esophageal aspirates of patients with GERD.^10^, ^13^ We used full growth medium (Dulbecco modified Eagle's medium/F12 10% FBS, 1% pen/strep, Gibco^®^, NY), brought to a pH of 4.0 with 1 M HCl (using a pH meter), (b) bile at pH 5.5 (weakly acidic), containing the same bile salts mixture in DMEM/F12 10% FBS, brought to a pH of 5.5 with 1 M HCl (using a pH meter), and (c) bile at pH 7.0, containing the same bile salts mixture in DMEM/F12 10% FBS, at pH 7.0.^17^, ^18^, ^19^, ^20^, ^21^, ^22^, ^23^

Control groups included repetitive exposure to (a) acid control, full growth DMEM/F12 10% FBS, brought to pH 4.0 with 1 M HCl, (b) weakly acidic control, full growth DMEM/F12 10% FBS, brought to pH 5.5 with 1 M HCl, and (c) neutral control, full growth DMEM/F12 10% FBS, pH 7.0.

After each application we removed the experimental and control media and cells were incubated in serum free media (Human Hypopharyngeal Normal Cell Culture Media Serum Free), for HHPC cells; (Celprogen Inc.), until the next application. Cells were harvested at the end of the treatment cycle (Day #4).

### 2.2 Immunofluorescence assay

Immunofluorescence assay was performed to detect (a) NF‐κB activation (p‐p65 S536), and (b) STAT3 activation (p‐STAT3 Tyr705), as previously described^17^, ^20^, ^21^ and in supplementary methods. HHPCs underwent repetitive exposures for 7 minutes to primary bile acids bile with DCA at pH 4.0, 5.5 or 7.0 respectively and in parallel to corresponding controls at pH 4.0. 5.5 and 7.0, as described above.

### 2.3 Western blotting

Western blot analysis was used to determine the protein expression levels of p‐NF‐κB (p65 S536) in nuclear and cytoplasmic extracts, as well as of p‐IKB‐α (S32/S36) and bcl‐2 in cytoplasmic extracts, of experimental and control groups, as has been previously described^17^, ^19^, ^21^, ^22^, ^23^ and in supplementary methods. We used β‐actin and Histone 1 for cytoplasmic nuclear extracts normalization while expression levels were estimated using Image Lab 5.2 analysis software (BIO‐RAD).

### 2.4 Luciferase assay

Luciferase assay was used to identify the NF‐κB transcriptional activity in hypopharyngeal cells under bile exposure at each pH point. We used pGL4.32[luc2P/NF‐κB‐RE/Hygro] Vector for NF‐κB reporter and (pGL4.27[luc2P/minP/Hygro]) as a control vector, a firefly Luciferase Assay system (Promega Corporation, Madison, WI), and Lipofectamine^®^ 2000 (Invitrogen^™^), as described previously.^22^ Briefly, equal number of cells were transfected with NF‐κB (NF‐κB‐Luc2P) or control (Luc2P) luciferase vector. We performed triplicate assays for each treatment condition 24 hours after transfection: exposure of HHPC in parallel for 7 minutes to bile mixture with or without DCA at pH 4.0, 5.5 and 7.0, DCA alone at pH 4.0, 5.5, 7.0 and corresponding controls at pH 4.0, 5.5, 7.0. At the end of the treatments, we measured luminescence using a luminometer (Infinite^®^ M1000 PRO, TECAN) and i‐control^™^ software. We expressed NF‐κB activity as ratios of mean values [values for NF‐κB reporter (NF‐κB‐Luc2P), against mean values for control (Luc2P) calculated in treated HHPC for each condition.

### 2.5 Quantitative real‐time PCR

Total RNA was isolated (RNeasy mini kit; Qiagen Inc., CA) from experimental groups, treated by primary bile acids with DCA at pH 4.0, 5.5, and 7.0, and corresponding controls at pH 4.0, 5.5 and 7.0. We used real‐time quantitative polymerase chain reaction (qPCR) analysis to evaluate the mRNA levels of *RELA(p65)*,* c‐REL*,* bcl‐2*,* TNF‐*α, *EGFR*,* STAT3* and *WNT5A*, as we have previously described in our prior explorations^19^, ^21^, ^22^, ^23^ and described in supplementary methods. Data were analyzed from three independent experiments using CFX96 Manager^™^ software (BIO‐RAD).

### 2.6 NF‐κB signaling pathway

PCR microarray analysis was used to investigate the NF‐κB signaling pathway in groups treated with a bile mixture of primary bile acids and secondary bile acid DCA vs control at strongly acidic pH. We used HHPC transcriptome and RT^2^‐Profiler PCR array, PAHS‐025z (SABiosciences, Qiagen), according to the manufacturer's instructions. The data analysis was performed using RT^2^‐Profiler PCR Array Data Analysis version 3.5 software. Up‐ or down‐regulation was assigned as >2‐fold change in gene expression between the experimental group (bile with DCA at pH 4.0; Group 1) and control‐treated group (Control).

### 2.7 Statistical analysis

We used GraphPad Prism 7 software for our statistical analysis and one‐way ANOVA (Friedman; *P* < 0.01) and multiple *t*‐test (by Holm‐Sidak) to compare the protein and mRNA expression between the experimental and control groups. Pearson correlation (*P* < 0.05) was used to estimate the correlation coefficient between expression levels among the different groups.

## 3 RESULTS

### 3.1 pH 4.0 optimally enhances the bile‐induced NF‐κB and STAT3 activation and bcl‐2 overexpression

**Figure 1:**
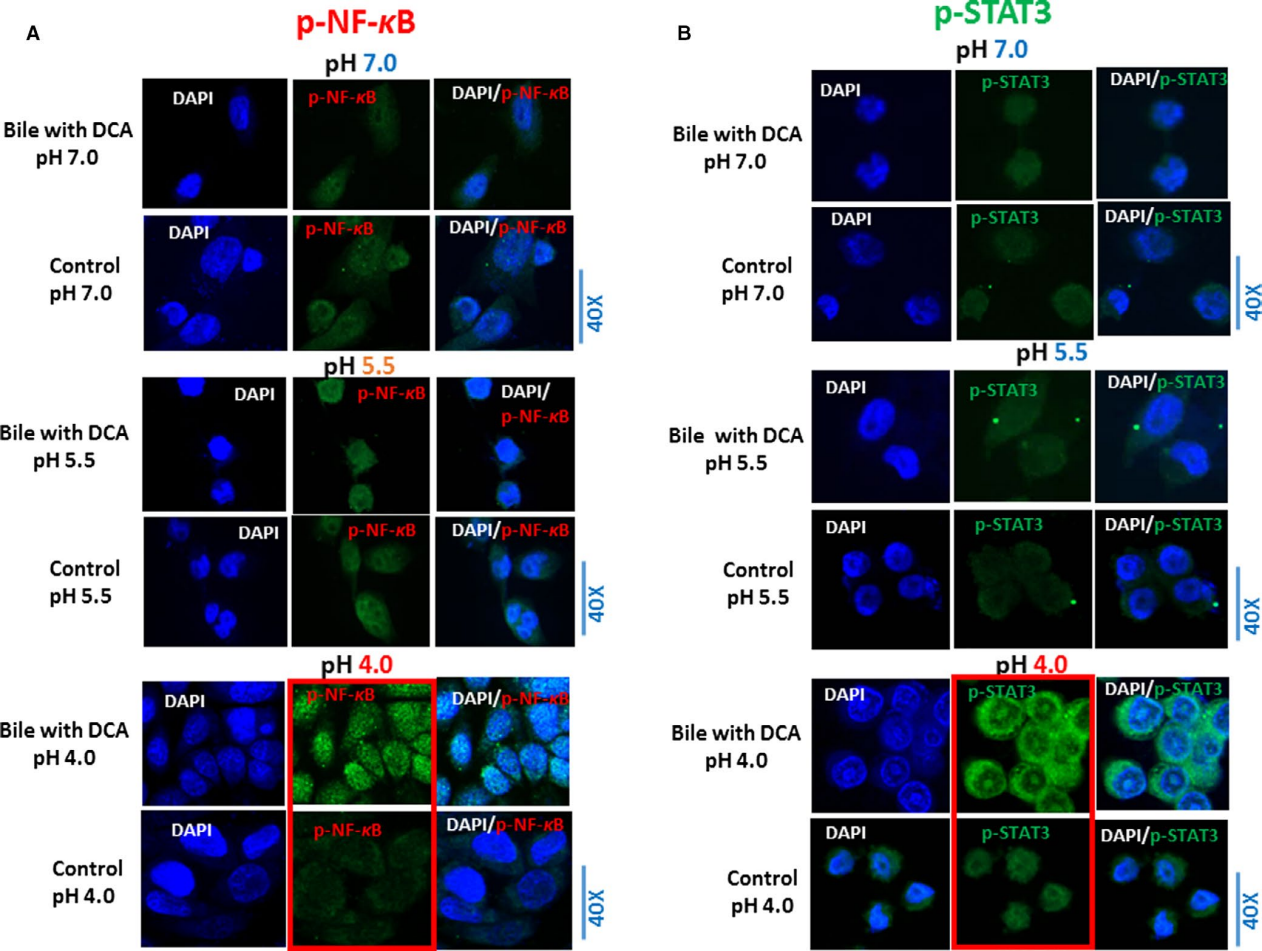
pH 4.0 optimally enhances bile‐induced nuclear translocation of phospho‐NF‐κB and activation of STAT3 in HHPC. Immunofluorescence staining for phospho‐NF‐κB (p‐p65 S536) reveals that primary bile acids with DCA at strongly acidic pH (4.0) induced (A) p‐NF‐κB nuclear translocation, demonstrating increased p‐NF‐κB nuclear staining [green: p‐p65 (S536); blue: DAPI for nuclear staining] and (B) activation of STAT3, demonstrating elevated phospho‐STAT3(Tyr707) staining [green: p‐STAT3 (Tyr707); blue: DAPI for nuclear staining], compared to HHPC exposed to bile at weakly acidic (5.5) or neutral pH (7.0) and controls at pH 4.0. 5.5 and 7.0. DCA, deoxycholic acid; HHPC, human hypopharyngeal primary cells

By immunofluorescence, pH 4.0 induced the most intense NF‐κB nuclear translocation in HHPC treated with bile (primary bile acids with DCA) compared to treatments at pH 5.5 or 7.0 (Figure 1A). HHPC treated with bile at strongly acidic pH (4.0) showed a more intense nuclear p‐ NF‐κB (p65 S536) staining compared to control at pH 4.0, 5.5 and 7.0. HHPC treated with bile at neutral pH (7.0) demonstrated weak cytoplasmic and nuclear staining of NF‐κB similar to its corresponding control (pH 7.0). However, HHPC exposed to bile at weakly acidic pH 5.5 presented a moderate p‐NF‐κB nuclear and cytoplasmic staining compared to its corresponding control (pH 5.5), as well as compared to cells exposed to neutral bile (pH 7.0).

We observed that pH 4.0 induced the most intense p‐STAT3 (Tyr705) staining in bile (primary bile acids with DCA) treated HHPC relative to other experimental and control groups (Figure 1B). Cells exposed to bile at strongly acidic pH (4.0) demonstrated a more pronounced nuclear and cytoplasmic p‐STAT3 (Tyr705) stain compared to those exposed to bile at weakly acidic or neutral pH (5.5 or 7.0, respectively) and to related controls.

**Figure 2:**
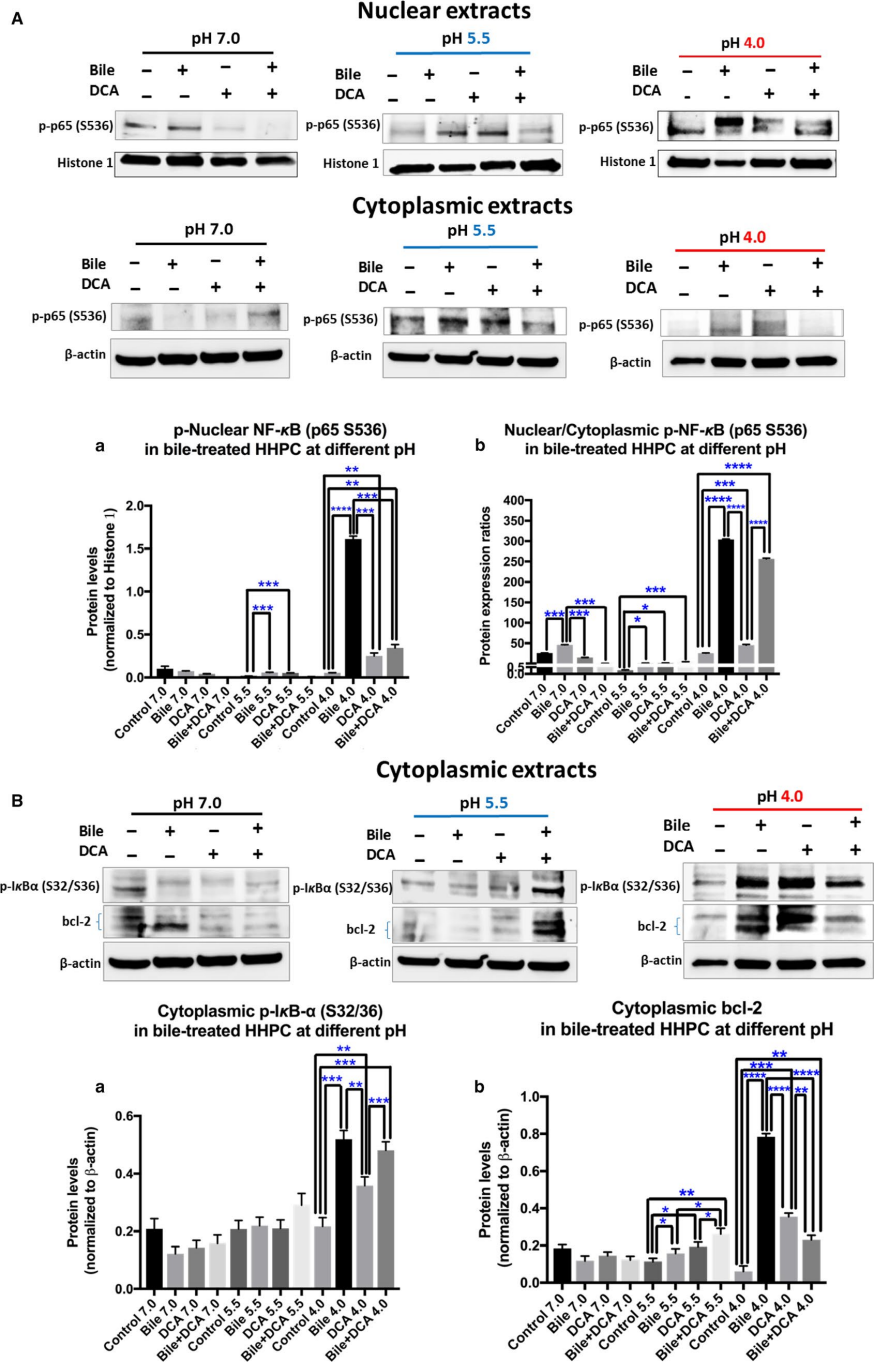
pH 4.0 optimally enhances bile‐induced NF‐κB activation and bcl‐2 overexpression in HHPC. Western blot analysis is performed in nuclear and cytoplasmic protein extracts of HHPC for p‐NF‐κB (p65 S529), and cytoplasmic p‐IKB‐α and bcl‐2. Strongly acidic pH (4.0) induced the highest bile‐induced (Α) (a) nuclear p‐p65 levels, (b) nuclear translocation (nuclear/cytoplasmic) p‐NF‐κB ratios, (B) (a) cytoplasmic p‐IKB‐α (S32/36) and (b) bcl‐2 levels, in treated HHPC compared to weakly acidic (5.5) or neutral pH (7.0). Specifically, HHPC exposed to bile (primary bile acids) at pH 4.0, 5.5 or 7.0 induced higher (A‐a) nuclear p‐p65 compared to bile with DCA and controls at pH 4.0, 5.5 and 7.0, respectively. HHPC exposed to primary bile acids with DCA at pH 4.0 or 5.5 induced significantly higher (A‐b) nuclear translocation (nuclear/cytoplasmic) p‐NF‐κB ratios compared to controls at pH 4.0 and pH (5.5), respectively. HHPC exposed to bile with or without DCA at pH 4.0 or pH 5.5 produced higher cytoplasmic (B‐a) p‐IKB‐α and (B‐b) bcl‐2 levels compared to DCA or control at pH 4.0 (Paired *t*‐test, *P < 0.05; **P <* *0.005; ***P < 0.0005; ****P < 0.00005; GraphPad Prism 7.0) (Mean ± SD of three independent experiments) (β‐actin and Histone 1 are used for the normalization of cytoplasmic and nuclear protein extracts, respectively). DCA, deoxycholic acid; HHPC, human hypopharyngeal primary cells

In general, western blot analysis confirmed the above immunofluorescence observations. Strongly acidic pH was more effective in inducing bile‐induced NF‐κB activation compared to weakly acidic or neutral conditions (Figure 2). Figure 2 shows that pH 4.0 induced the highest p‐NF‐κB nuclear translocation (Figure 2A). HHPC treated with strongly acidic bile (primary bile acids with DCA) produced significantly higher nuclear p‐NF‐κB levels (Figure 2A‐a) and nuclear p‐NF‐κB translocation ratios (Figure 2A‐b), relative to acid alone (*P *<* *0.005 and *P *<* *0.00005, respectively). At weakly acidic pH (5.5) bile induced mildly higher p‐NF‐κB nuclear translocation ratios, compared to corresponding control (*P *<* *0.0005).

In addition, strongly acidic pH (4.0) induced higher p‐IKB‐α cytoplasmic levels in bile (primary bile acids with or without DCA) treated cells, relative to other experimental or controls groups at weakly acidic or neutral pH (5.5 or 7.0, respectively) (Figure 2B‐a). Cytoplasmic p‐IKB‐α levels were found to be significantly more elevated in HHPC exposed to strongly acidic bile (primary bile acids with or without DCA) compared to acid alone (pH 4.0).

Western blot analysis on cytoplasmic extracts of experimental and control groups across pH levels revealed the effect of each bile mixture (primary bile acids with or without DCA) on bcl‐2. We found that strongly and weakly acidic pH (4.0 and 5.5) induced the highest levels of bcl‐2 levels in cells exposed to bile (primary bile acids with or without DCA), with statistically significant differences relative to corresponding controls (Figure 2B‐b).

Pearson analysis revealed a significant positive correlation between cytoplasmic p‐IKB‐α and nuclear p‐p65 levels (*r *=* *0.77, *P *=* *0.002) or p‐p65 nuclear translocation ratios (*r *=* *0.87, *P *=* *0.0001). A significant positive correlation was also identified between p‐IKB‐α and bcl‐2 cytoplasmic levels (*r *=* *0.79, *P *=* *0.001).

### 3.2 pH effect of primary bile acids with and without secondary bile acid (DCA) in NF‐κB activation and bcl‐2 overexpression

The effect of strongly acidic pH (4.0) in activating NF‐κB and inducing bcl‐2 overexpression was found to be more pronounced in HHPC exposed to primary bile acids without DCA (Figure 2A). In contrast, weakly acidic pH (5.5) was more effective in activating NF‐κB and inducing bcl‐2 overexpression in HHPC exposed to primary bile acids with DCA (Figure 2B).

Specifically, the effect of strongly acidic pH (4.0) was found to be a critical factor for nuclear translocation of p‐NF‐κB in cells treated with conjugated primary bile acids without secondary bile acid DCA, especially when compared to bile containing DCA (Figure 2A‐b). However, weakly acidic pH (5.5) was found more effective in p‐NF‐κB nuclear translocation in primary bile salts containing DCA compared to primary bile acids alone, whereas at pH 7.0 p‐NF‐κB nuclear translocation was higher in primary bile acids alone vs primary bile acids with DCA or DCA alone (Figure 2A‐b).

The effect of a strongly acidic mixture of conjugated primary bile acids without DCA at pH 4.0 was most effective in inducing elevated p‐IKB‐α levels relative to other groups, with a statistically significant difference compared to acid alone (Figure 2B‐a).

We also found that strongly acidic pH contributed to bcl‐2 overexpression in HHPC exposed to mixtures of conjugated primary bile acids, producing higher bcl‐2 levels compared to primary bile acids with DCA and controls at pH 4.0 (Figure 2B‐b). On the other hand, weakly acidic pH (5.5) contributed to a significant bcl‐2 overexpression in cells treated with primary bile acids with DCA relative to primary bile acids without DCA and corresponding weakly acidic control pH (5.5).

### 3.3 NF‐κB transcriptional activity induced by the effect of bile is optimized at pH 4.0

**Figure 3:**
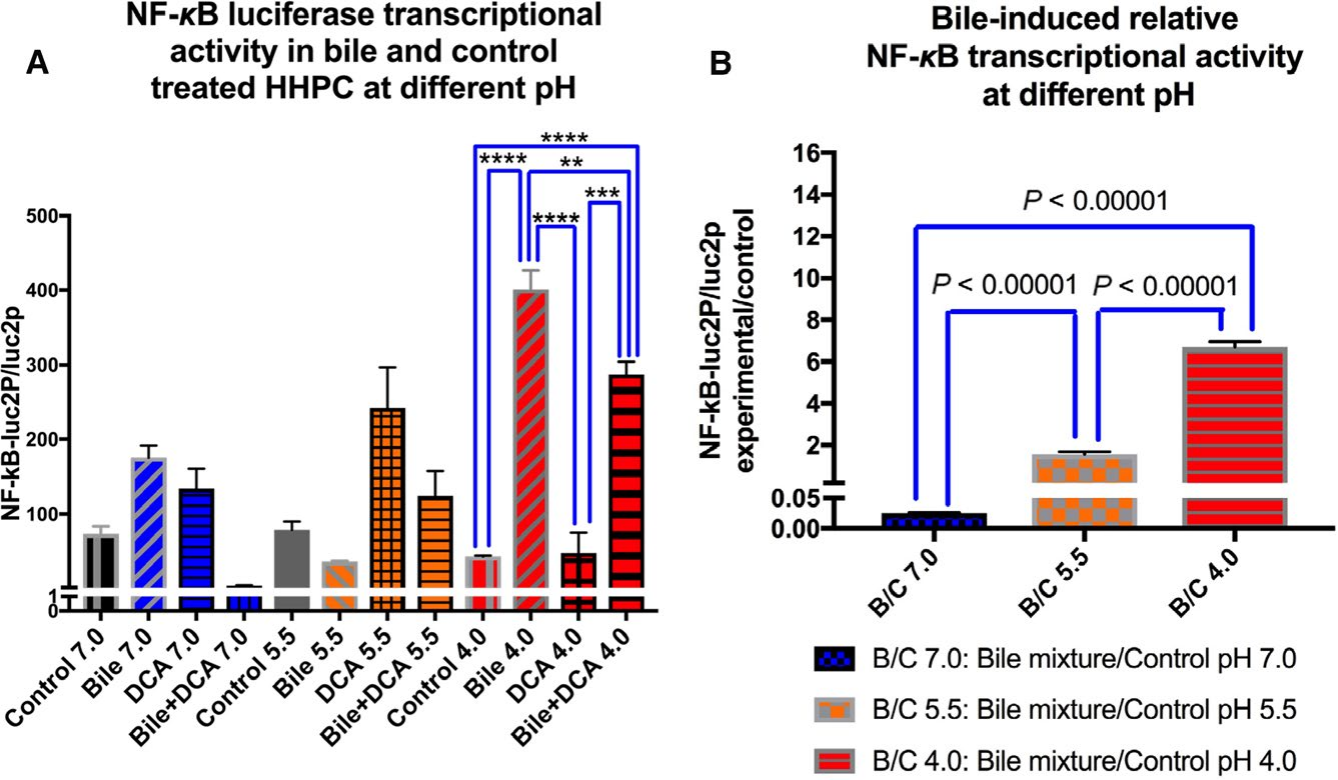
Bile‐induced NF‐κB transcriptional activity is optimized at pH 4.0 in HHPC. A, Columns represent luciferase NF‐κB relative transcriptional activity (mean ± SE of two independent experiments) in HHPC transfected with control luciferase reporter (luc2P) and NF‐κB luciferase responsive element (NF‐κB‐Luc2P). B, Columns represent ratios of luciferase NF‐κB relative transcriptional activity between experimental groups (primary bile acids with DCA) and controls at pH 4.0, 5.5 and 7.0 (NF‐κB‐Luc2P/Luc2P: NF‐κB luciferase responsive element/control luciferase reporter). DCA, deoxycholic acid; HHPC, human hypopharyngeal primary cells

The luciferase assay revealed that strongly acidic pH (4.0) was a critical factor for maximum NF‐κB transcriptional activity (Figure 3A). Weakly acidic pH (5.5) could also affect the primary bile acids with DCA to activate transcriptionally NF‐κB. However, the luciferase assay showed that HHPC treated with primary bile acids with or without DCA at strongly acidic pH (4.0) induced the highest transcriptional activity of NF‐κB relative to other experimental and control groups, with statistically significant differences compared to control or DCA alone at pH 4.0 (Figure 3A).

Figure 3B shows the relative NF‐κB transcriptional activity between primary bile acids with DCA and corresponding control groups, at strongly acidic, weakly acidic and neutral pH. Strongly acidic pH (4.0) was found to be most effective in inducing transcriptional activity of NF‐κB in cells treated with bile, demonstrating significantly higher ratios of experimental to control NF‐κB transcriptional activity (NF‐κB reporter [NF‐κB‐Luc2P], against control [Luc2P]) compared to weakly acidic or neutral pH. Likewise, weakly acidic pH induced higher ratios of relative transcriptional activity of NF‐κB in bile‐treated cells compared to neutral pH (Figure 3B).

The effect of strongly acidic pH on NF‐κB transcriptional activity was found to be more pronounced in HHPC exposed to conjugated primary bile acids alone vs primary bile acids with the secondary bile acid DCA (Figure 3A).

### 3.4 Bile‐induced transcriptional activation of cancer‐related genes is optimized at pH 4.0

**Figure 4:**
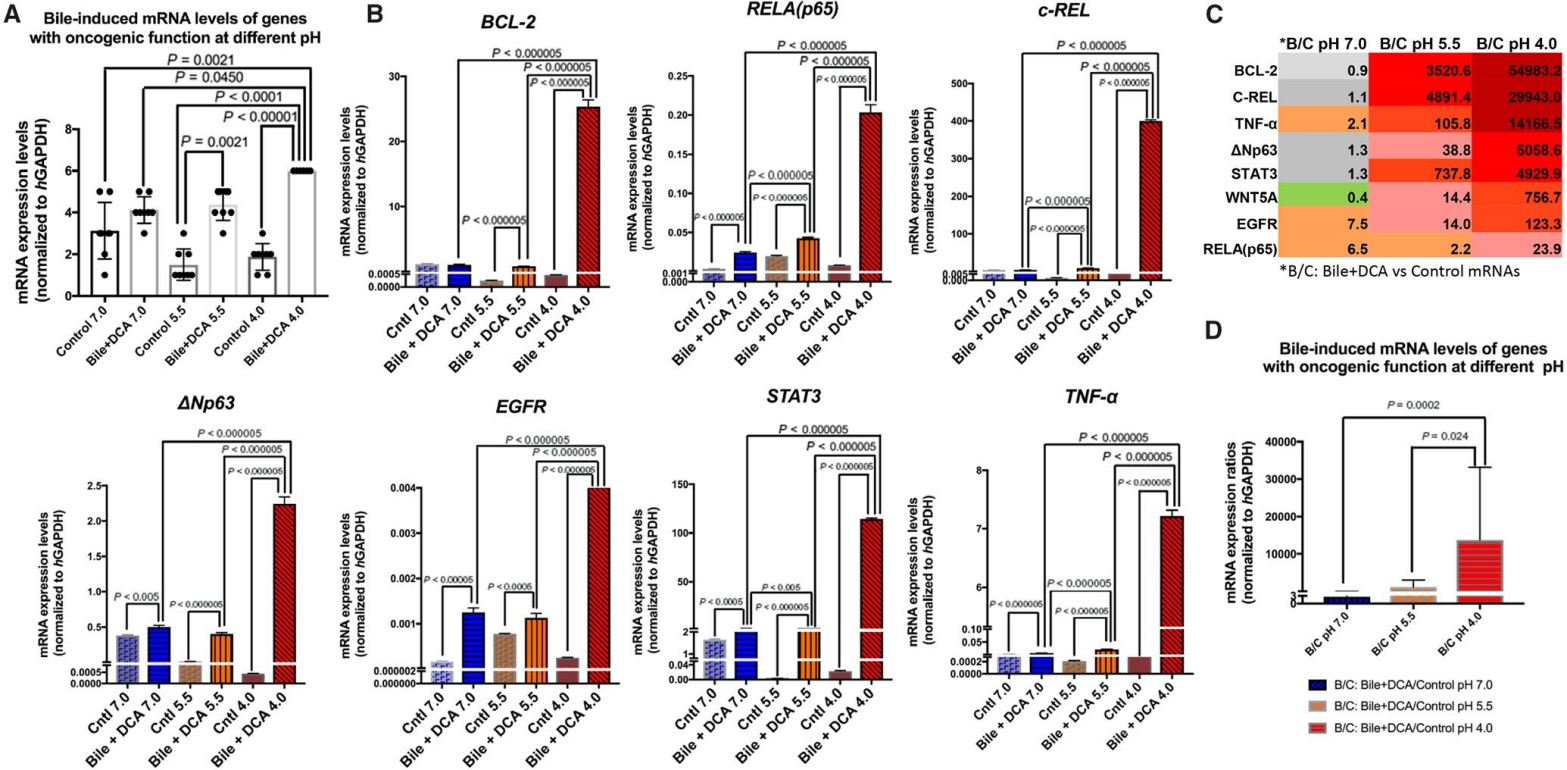
Bile‐induced transcriptional activation of cancer‐related genes is optimized at pH 4.0 in HHPC. A, The highest transcriptional levels of the analyzed NF‐κB‐related genes with oncogenic function, are depicted in HHPC exposed to primary bile acids with DCA at strongly acidic pH (4.0) relative to HHPC exposed to bile with DCA at weakly acidic (5.5) or neutral pH (7.0) and controls at pH 4.0, 5.5 and 7.0. Weakly acidic pH (5.5) also induced higher transcriptional level of the analyzed genes compared to corresponding control at pH 5.5. Graphs, created by GraphPad Prism 7 software (transcriptional levels of the analyzed genes are normalized to hGAPDH by real‐time qPCR analysis) (one‐way ANOVA, Freidman test). B, Graphs represent transcriptional levels of each analyzed gene, *RELA(p65)*,* TNF‐*α, *STAT3*,* bcl‐2*,* WNT5A*, and *EGFR* (relative to hGAPDH reference gene), in experimental and control‐treated HHPC. The data are derived from real‐time qPCR analysis. C, Table shows overexpression ratios between bile (primary bile acids) with DCA at pH 4.0, 5.5 and 7.0 and corresponding controls (data are derived from three independent experiments. Graphs, created by GraphPad Prism 7 software; by *t‐*test; multiple comparisons by Holm‐Sidak). DCA, deoxycholic acid; HHPC, human hypopharyngeal primary cells

In general, HHPC exposed to primary bile acids with DCA at strongly acidic pH 4.0 produced the highest mRNA levels of all studied genes with a statistically significant difference compared to controls at pH 4.0, 5.5 and 7.0, as well as compared to primary bile acids with DCA at neutral pH (7.0) (Figure 4A). Bile with DCA at weakly acidic pH 5.5 also induced statistically higher mRNA levels of the studied genes relative to its corresponding control at pH 5.5 (Figure 4A).

qPCR revealed that HHPC exposed to primary bile acids with DCA at pH 4.0 exhibited a significant overexpression of the anti‐apoptotic *bcl‐2*, NF‐κB transcriptional factors *RELA(p65)*, and *c‐REL*, and oncogenic *EGFR*,* STAT3*,* TNF‐*α and *ΔNp63*, with a significant difference relative to bile with DCA at weakly acidic or neutral pH (Figure 4B,C).

Although, weakly acidic pH (5.5) was less effective than strongly acidic pH (4.0) in inducing transcriptional activation of the analyzed genes in cells treated with primary bile acids with DCA, it was capable of inducing significantly higher mRNAs of *RELA(p65)*,* c‐REL*,* TNF‐*α and *STAT3* relative to bile with DCA at neutral pH (7.0) (Figure 4B,C).

Primary bile acids with DCA at neutral pH (7.0) were found less effective than bile at weakly acidic pH in inducing transcriptional activation of the analyzed genes (Figure 4B,D). However, neutral bile was found capable of inducing overexpression of *RELA(p65)*,* TNF‐*α, *STAT3*,* EGFR* and *ΔNp63* with significantly higher mRNAs compared to its corresponding control (Figure 4B,C).

### 3.5 Strong positive correlations among bile‐induced transcriptional levels of NF‐κB‐related genes

A Pearson analysis revealed significant linear correlations among mRNA levels of the studied genes at pH points 4.0, 5.5 and 7.0.

We found strong positive correlations between NF‐κB transcriptional factor *RELA(p65)* and (a) *STAT3*,* EGFR*,* TNF‐*α, *bcl‐2* (*r *>* *0.98, *P *<* *0.0001); (b) *ΔNp63*,* c‐REL* (*r *>* *0.97, *P *<* *0.0003); (c) *WNT5Α* (*r *>* *0.86, *P *<* *0.05).

We also observed significant positive correlations between transcriptional factor *STAT3* and (a) *EGFR*,* TNF‐*α, *bcl‐2* (*r *>* *0.86, *P *<* *0.05); (b) *ΔNp63*,* c‐REL* (*r *>* *0.97, *P *<* *0.0002), (c) *WNT5Α* (*r *>* *0.88, *P *<* *0.01).

Significant positive correlations were identified between growth factor *EGFR* and (a) *TNF‐*α, *bcl‐2* (*r *>* *0.1, *P *<* *0.0001); (b) *ΔNp63*,* c‐REL* (*r *>* *0.97, *P *<* *0.0002); (c) *WNT5Α* (*r *>* *0.88, *P *<* *0.01). We also found significant positive correlations between cancer‐related cytokine *TNF‐*α and (a) *bcl‐2* (*r *>* *0.1, *P *<* *0.0001); (b) *ΔNp63*,* c‐REL* (*r *>* *0.97, *P *<* *0.0003); (c) *WNT5A* (*r *>* *0.88, *P *<* *0.01).

We observed significant positive correlations between cell proliferation factor *ΔNp63* and (a) *bcl‐2* (*r *>* *0.98, *P *<* *0.0001), (b) *c‐REL* (*r *>* *0.97, *P *<* *0.0003), (c) *WNT5Α* (*r *>* *0.94, *P *<* *0.005). Finally, significant positive correlations were found between NF‐κB transcriptional factor *c‐REL* and (a) *bcl‐2* (*r *>* *0.1, *P *<* *0.0001), (b) *WNT5Α* (*r *>* *0.88, *P *<* *0.01) and between anti‐apoptotic *bcl‐2* and (c) *WNT5Α* (*r *>* *0.9, *P *<* *0.01).

A significant positive correlation was also found between nuclear p‐NF‐κB protein levels and mRNA levels of NF‐κB transcriptional factor *RELA(p65)* (*r *>* *0.9, *P *<* *0.005).

### 3.6 A mixture of bile including primary bile acids and the secondary bile acid, DCA, at strongly acidic pH, induced upregulation of the NF‐κB signaling pathway

Strongly acidic pH (4.0) bile mixtures of primary bile acids including DCA, induced the highest levels of NF‐κB‐related mRNA oncogenic phenotype compared to weakly or neutral pH. We used a PCR array to explore how primary bile acids with DCA at strongly acidic pH (pH 4.0) could up‐regulate the expression of genes of NF‐κB signaling when compared to controls. We observed that primary bile acids together with DCA at pH 4.0 produced upregulation of 39 out of 84 analyzed NF‐κB‐related genes (~46%) (>2‐fold change) (Table 1).

**Table 1:** Up‐ or down‐regulated genes of NF‐κB signaling in human hypopharyngeal primary cells under exposure to acidic bile mixture of conjugated bile acids including secondary unconjugated bile acid, deoxycholic acid

Gene symbol	DBA/Cntl^a^	Gene symbol	DBA/Cntl	Gene symbol	DBA/Cntl
AGT	4.4	HMOX1	2.65	REL	1.2
AKT1	−18.45	ICAM1	2.14	RELA	4.07
ATF1	−4.54	IFNA1	−1.25	RELB	−3.28
BCL10	1.2	IFNG	1.14	RHOA	5.59
BCL2A1	−1.16	IKBKB	4.05	RIPK1	2.96
BCL2L1	−1.5	IKBKE	14.76	STAT1	−1.7
BCL3	3.09	IKBKG	−147.56	TBK1	1.78
BIRC2	3.04	IL10	11.65	TICAM1	1.34
BIRC3	22.44	IL1A	3.14	TICAM2	−1.25
CARD11	11.1	IL1B	−1.25	TIMP1	−1.46
CASP1	2.09	IL1R1	−1.25	TLR1	1.4
CASP8	−1.25	CXCL8	2.66	TLR2	−1.25
CCL2	−1.05	IRAK1	−11.33	TLR3	4.03
CCL5	5.69	IRAK2	−17.46	TLR4	2.46
CD27	−5.82	IRF1	5.15	TLR6	12.75
CD40	1.1	JUN	−12.64	TLR9	1.66
CFLAR	1.14	LTA	3.74	TNF	−1.25
CHUK	−2.34	LTBR	2.31	TNFAIP3	−1.25
CSF1	7.19	MALT1	3.31	TNFRSF10A	−1.25
CSF2	1.06	MAP3K1	1.06	TNFRSF10B	7.43
CSF3	−2.32	MYD88	−2.61	TNFRSF1A	4.41
EGFR	−1.69	NFKB1	−3.27	TNFSF10	11.53
EGR1	12.59	NFKB2	2.08	TNFSF14	46.94
ELK1	3.52	NFKBIA	2.01	TRADD	3.81
F2R	2.05	NFKBIE	−1.25	TRAF2	−1.7
FADD	−1.19	NOD1	6.5	TRAF3	8.13
FASLG	11.29	PSIP1	−1.25	TRAF6	10.45
FOS	−33.69	RAF1	1.47		

a DBA/Cntl: relative normalized mRNA expression ratios (acidic bile mixture vs control).

The effect of primary bile acids together with DCA on the NF‐κB pathway is provided in Table 1. Bile with DCA induced upregulation of the mRNA levels of the NF‐κB transcription factors, *RELA(p65)* (4‐fold), and *NF‐*κ*B2* (>2‐fold), as well as members of TNF‐receptors, such as *TNFRSF10B* (>7‐fold), *TNFRSF1A* (>4‐fold), *TNFSF10* (>11‐fold) and *TNFSF14* (>46‐fold). Bile with DCA at pH 4.0 also induced the upregulation of the transcriptional levels of receptors and ligands of the innate immune system, such as *TLR3* (>4‐fold), *TLR4* (2.5‐fold) *TLR6* (>12‐fold), *IL1A* (>3‐fold) and others. Bile with DCA induced upregulation of NF‐κB downstream signaling, producing the expression of positive regulators of the NF‐κB pathway, such as *BIRC2* (>3‐fold), *IRF1* (>5‐fold), LTA (>3.5‐fold), *TRAF3* (>8‐fold) and *TRAF6* (>10‐fold). Bile with DCA produced the upregulation of Inhibitor‐kappaB kinases, *IKBKB* (>4‐fold) and *IKBE* (>14‐fold), as well as of *BCL‐3* (>3‐fold), which is a coactivator of NF‐κB, inducing the cytoplasmic release of NF‐κB. Together we observed an increase in the expression of anti‐apoptotic genes, such as *BIRC2* (>3‐fold) and *BIRC3* (>22‐fold) genes.

Bile with DCA at pH 4.0 resulted in upregulation of many NF‐κB responsive genes, enhancing the production of transcriptional factors, such as *EGR1* (>12‐fold) and *ELK1* (>3.5‐fold). We also found an upregulation of *CARD11* (>11‐fold), activator of NF‐κB through *BCL10*. Finally, primary bile acids with DCA activated other NF‐κB signaling genes, such as *Hmox1* (>2.6‐fold) which has a protumorigenic role.

## 4 DISCUSSION

In nonsmokers, acid reflux is considered an independent risk factor in laryngopharyngeal carcinogenesis, in a role similar to gastroesophageal reflux in the development of Barrett's lower esophagus and esophageal neoplasia. Lewin et al., found a high incidence (85%) of low pH (≤4.0) LPR among patients with premalignant and early laryngeal cancer.^24^ In contrast, Galli et al. suggested that biliary alkaline reflux might also be involved in the onset of laryngeal cancer.^25^ Interestingly, Langevin et al., showed an inverse association between antacid use and laryngopharyngeal carcinoma in patients with a history of heartburn, relative to those never taking heartburn medication, suggesting a protective effect of antacid medications.^5^ Other epidemiologic evidence suggests that head and neck cancer patients using antacid therapy have a more favorable outcome.^26^ As a result, the importance of the pH in the development and promotion of malignancies of the upper aerodigestive tract and the effectiveness of antacids in disease prevention remains unclear. The exploration of how pH affects molecular changes related to hypopharyngeal carcinogenesis will not only contribute to a clarification of key aspects of the disease's pathophysiology, but will also exert clinical influence in determining the importance of antacid therapy for the prevention of reflux‐related hypopharyngeal cancer. It is hoped that the data presented in this study contribute to an improved understanding of the pathophysiology and potential therapies related to laryngopharyngeal carcinogenesis.

With a glycine‐to‐taurine conjugate ratio of 3:1, glycine‐conjugated bile acids are the predominant bile acids aspirated from the esophagus of patients with reflux.^27^ However, at strongly acidic pH (<4.0) taurine‐conjugated bile acids are ionized and therefore less soluble than glycine‐conjugated bile acids. At weakly acidic pH (5.0‐5.5) a proportion of glycine‐conjugated bile acids may remain un‐ionized and therefore capable of interacting with cell membranes, similar to their unconjugated counterparts at these conditions. For example, DCA, an unconjugated secondary bile is un‐ionized at pH 5.5 and therefore preferentially capable of interacting with the cell membrane in this pH range.

Our novel findings document an overall increase in the biliary reflux‐related tumorigenic effect on hypopharyngeal cells as the pH falls. We show that bile at strongly or even weakly acidic pH increases NF‐κB transcriptional activity and promotes the overexpression of cancer‐related genes, supporting a bile‐related oncogenic effect between pH 4.0 and 5.5. We further demonstrate that bile exerts its most harmful effect on HHPC at strongly acidic pH 4.0, inducing an intense transcriptional activation of NF‐κB transcriptional factors *RELA(p65)* and *c‐REL*, oncogenic *STAT3* and *TNF‐*α, as well as *EGFR*,* ΔNp63*,* WNT5Α* and anti‐apoptotic *bcl‐2*.

Our data support the understanding that the pH dependent effect of bile is closely related to its composition. Taking into consideration that taurine conjugates are active at low pH (<4.0) it is likely that these components are more responsible for the described NF‐κB‐related oncogenic response at lower pH. On the other hand, since glycine‐conjugated bile acids may be partially active at pH 5.5, similar to unconjugated DCA, it is likely that both are potent activators of NF‐κB‐related oncogenic pathway at weakly acidic environment, such as pH 5.5.

In conclusion, our findings strongly suggest that a biliary tumorigenic effect on hypopharyngeal cells is significantly potentiated by pH reduction. The activated levels of NF‐κB and its related downstream anti‐apoptotic and oncogenic pathways are positively related to the acidity of bile. Neutral pH is less effective than weakly acidic pH, and weakly acidic pH is less effective than strongly acidic pH in activating the bile‐related tumorigenic effect. The increased bile‐induced tumorigenic effect during reflux events as the pH drops to 4.0 may be due to the activation of primary bile acids and their interactions with the cell membrane. As pH grows less acidic approaching pH 5.5 the partially activated primary bile acids and the activated secondary bile acids, such as DCA, exert their influence. Controlling pH during reflux episodes may have a therapeutically protective effect on the risk for bile‐induced hypopharyngeal cancer and may be important in the prevention of cancer recurrence or prevention of second primary cancers in at risk populations.

## DISCLOSURE

The authors whose names are listed in this article certify that they have NO affiliations with or involvement in any organization or entity with any financial interest, or nonfinancial interest in the subject matter or materials discussed in this manuscript.
