# Supplementary material for: Biliary tumorigenic effect on hypopharyngeal cells is significantly enhanced by pH reduction
Source: Cancer Med. 2019 Jun 7;8(9):4417–27. doi: 10.1002/cam4.2194 (PMC6675744; doi:10.1002/cam4.2194)
Supplement: Supplementary file 1 [file CAM4-8-4417-s001.docx]

**Supplementary information**

**Title: Biliary tumorigenic effect on hypopharyngeal cells is significantly enhanced by pH reduction**

**Supplement to Methods**

***Immunofluorescence assay:*** HHPCs were grown on slides (multiwall chamber slides; Lab-Tek^®^) and underwent exposures for 7 min to primary bile acids bile with DCA at pH 4.0, 5.5 or 7.0 respectively and in parallel to corresponding controls, as described in “treatment conditions”.

At the end of treatment, cells were fixed immediately in 4% paraformaldehyde (Sigma-Aldrich) for 7 minutes and incubated overnight at 4°C with 1:65 of primary anti-NF-κB (rabbit polyclonal anti-phospho-p65 Ser536, AbD Serotec, BIO-RAD, CA, USA) and 1:100 of primary anti-phosho-STAT3 (Tyr705)(D3A7) XP Rabbit mAb, Cell signaling, Techn.), after permeabilization of cell membranes using 0.2% Triton X100 (AmericanBio, Natick, MA, USA) in PBS for 2-3 minutes and blocking with 2% bovine serum albumin (BSA) in PBS (Sigma-Aldrich, USA) for 1 hour. The next day, cells were first washed in 1% Tween20 (AmericanBio, Natick, MA, USA) in PBS for 1-2 min, then with 0.1% BSA in PBS twice for 5 min and subsequently incubated with 1:500 dilutions of secondary anti-rabbit DyLight^®^488 (green; Vector Labs, USA), for 1 hour, at room temperature, washed and mounted using Prolong Gold Mountant with diamidino-phenylindole (ProLong^®^ Diamond Antifade Mountant with DAPI; Life Technologies, Thermo Scientific, MA, USA) for nuclear staining and mounting of cells (blue). Slides were examined using a Zeiss Confocal microscope and images were captured and analyzed using Zen imaging software from Carl Zeiss, microscopy (Germany).

***Western blotting:*** At the end of treatment 10 to 30 µg of HHPC nuclear and cytoplasmic protein extracts were heated at 70°C for 10 minutes in sodium dodecyl sulfate polyacrylamide gel electrophoresis Laemmli sample buffer (Bio-Rad, Hercules, CA), and were separated using 4-20% Mini-PROTEAN TGX Tris/Glycine pre-cast gels, at 150V for 1 hour, while Precision Plus Prestained Protein Standards (Dual Color or Kaleidoscope, *Bio-RAD*) were used providing a 10-band ladder (250-10 kD). Proteins were transferred onto a 0.45 mm nitrocellulose membrane, using Trans Blot Turbo transfer system (Bio-Rad), blocked in 5% BSA, for 1 hour, and were incubated with primary antibodies, anti-NF-kB (pS529) primary antibody, (Invitrogen^TM^, Thermo Fisher Scientific, Massachusetts, USA), phospho-IκB-α Ser32/36 (5A5; Cell Signaling, EMD Millipore, Billerica, MA), and bcl-2 (C-2; Santa Cruz Biotechnology), which were diluted in 5% BSA, overnight at 4 °C. Membranes were incubated for 1:30 hours with goat anti-rabbit or anti-mouse horseradish peroxidase conjugated secondary antibodies (EMD Millipore) at 1:5000 and chemiluminescence was determined using an enhanced chemiluminescence detection system (Clarity Western ECL Substrate, Bio-Rad). Membranes also were stripped using Restore stripping buffer (Pierce) and were reported with β-actin (C4; Santa Cruz Biotechnology) and Histone 1 (AE-4; Santa Cruz Biotechnology) for cytoplasmic nuclear extracts normalization. Protein levels were quantified by Gel imaging system (*BIO-RAD*). in each nuclear or cytoplasmic cellular compartment, and expression levels were estimated by Image Lab 5.2 analysis software (*BIO-RAD*).

**Quantitative real time PCR**

We determined RNA quality and concentration by absorption ratios at 260/280 nm (> 2.0) and 260 nm, respectively (NanoDrop™ 1000 spectrophotometer; Thermo Fisher Scientific, Waltham, MA). We performed reverse transcription (iScript cDNA synthesis kit; Bio-Rad) and real time qPCR analysis (Bio-Rad real time thermal cycler CFX96^TM^; Bio-Rad) using specific primers for target genes and reference housekeeping gene, human glyceraldehyde 3-phosphate dehydrogenase (*h*GAPDH), (QuantiTect Primers Assays; Qiagen) (Table S1), and iQ™ SYBR Green Supermix (Bio-Rad). We performed assays in 96-well plates, in triplicate for each sample, and data were analyzed by CFX96™ software. Relative mRNA expression levels were estimated for each target gene relative to reference gene (ΔΔ*C*t). (Data were obtained from three independent experiments)

**Supplementary Table S1**: Human genes analyzed by real-time qPCR, in normal human hypopharyngeal cells.

| **Gene** | **Detected transcripts** | **Amplicon length (bp)** |
| --- | --- | --- |
| ***h*GAPDH** | NM_001256799, NM_002046 | 95 |
| **bcl-2** | NM_000633 | 116 |
| **EGFR** | NM_005228  NM_201282-4, | 80 |
| **REL** | NM_002908 | 117 |
| **RELA** | NM_001145138, NM_001243984-5, NM_021975 | 107 |
| **wnt5A** | NM_001256105, NM_003392 | 105 |
| **Tp63** | NM_001114980, NM_003722 | 130 |
| **TNF** | NM_000594 | 98 |
| **STAT3** | NM_003150,  NM_139276 | 95 |
| **IL-6** | NM_000600 XM_005249745 | 107 |
